# Abstract of the Proceedings of the Buffalo Medical Association

**Published:** 1878-05

**Authors:** Joseph Fowler

**Affiliations:** Secretary


ART. IV.—*Abstract of the Proceedings of the Buffalo Medical Association.* April 2, 1878.

The President, Dr. E. R. Barnes, in the chair. Present: Drs. Barnes, Johnson, Habenstreit, J. F. Miner, Jansen, Bartlett, McNeil, W. W. Miner, Little, Brecht, Hauenstein, Samo, Trowbridge, Lothrop, Rochester, Cronyn, Hopkins, Howe, Mynter, White and Fowler, and by invitation Dr. A. M. Barker.

The report of the Treasurer, Dr. Fowler, was presented, and on motion Drs. Hopkins and Cronyn were appointed a committee to audit his account.

Dr. White, the essayist for the evening, had entirely forgotten his appointment as such until a very late hour, and therefore was not prepared to present a carefully written paper. He said he would not disappoint the members, and proceeded extemporaneously with his subject, "The Forceps," directing his remarks more particularly to a description of the different instruments, their use and manner of application. In a few words the Doctor gave a brief historical sketch of the obstetric forceps as employed in midwifery, with many practical hints as to their use. (We shall publish Prof White's remarks in our next as a separate article.)

Dr. J. F. Miner, in discussing the paper, said that Dr. White had long taught young men how to use the forceps, and he believed very correctly. He thought they often failed by not understanding how to apply and manipulate them; he had repeatedly seen young men greatly mortified at not being able to use the forceps successfully. The difficulty often lies in the manner of attempted application, the handles not being depressed enough to bring the blades in proper relation with the head. Chloroform is proper to administer in instrumental délivery. You rarely observe any mothers perish from this process of labor, and it should be almost entirely unknown; but when death results it is from too great delay in their application.

Dr. Hauenstein thought that Dr. White had gone so ably over the ground that there was hardly anything he could add. He entirely agreed with him in the frequent use of the forceps when properly applied. Under such circumstances no harm can be done to either mother or child. He had been taught not to take instruments with him when called to attend cases of midwifery, as he might use them prematurely. He had learned from sad experience to carry his forceps with him to all labor cases; he once lost a patient from rupture of uterus., when her life might have been saved by the timely use of the forceps. Another difficulty of ap_ plication is in carrying the instrument in a direct line, the blade becoming fixed in the folds of the vagina. The blade should be kept in close contact with the hand while introducing it.

Dr. Bartlett felt under obligations to Dr. White for his ideas on this subject. It was his practice now to apply the forceps much oftener than formerly. Laceration to external parts was liable to occur if much traction be made just as the head was emerging from the lower straight. At this stage uterine contraction is very powerful, and much laceration might often be avoided by retarding the rapid advance of the head; he usually supports the perineum with one hand while manipulating the forceps with the other. He had recently restored a prolapsed funis in three cases by means of a little device consisting of two long pieces of whalebone, to one end of each was attached a narrow piece of tape about four inches in length; by placing these pieces parallel with each other a loop was formed capable of loosely grasping the cord, when it could be safely lodged in the cavity of the uterus.

Upon this point Dr. White said, that when the cord had descended and could not be carried up, and the circulation cut off, he would apply forceps immediately; he thought the device shown an ingenious one, but not superior to a long flexible catheter, with the eye of which a loop of tape that has already been loosely attached to the funis can be held securely by means of a stylet; the cord can then be introduced, and the stylet withdrawn, thus liberating the catheter.

Dr. Bartlett thought that it might be made useful when a catheter was not available.

Dr. White added, that the manner of application of the forceps should be upon the palmer surface of the hand. Dr. Flint and himself knew of a case where the blade was carried into the cavity of the abdomen outside of the uterus. Forceps should not be removed early, as they serve a useful purpose to retard too rapid progress.

After the conclusion of the discussion, the Auditing Committee reported that they had examined the vouchers of the Treasurer and found them correct. On motion, the report was received.

A mild form of scarlatina and influenza were reported under the head of prevailing diseases.

On motion, Drs. Wyckoff, Hopkins and J. F. Miner were appointed a committee to nominate officers for the ensuing year.

The committee reported the following nominations:

For President—T. Lothrop.
Vice-President—J. Hauenstein.
Secretary—Joseph Fowler.
Treasurer—F. E. L. Brecht.
Librarian—P. H. Strong.

On motion, the report of the committee was accepted, and the gentlemen named declared elected.

The Association then adjourned.

JOSEPH FOWLER, M. D.,
*Secretary.*

